# Accuracy of laparoscopy for assessing patients with endometriosis

**DOI:** 10.1590/S1516-31802008000600002

**Published:** 2008-11-06

**Authors:** Dilermando Pereira de Almeida, Laerte Justino de Oliveira, Vivian Ferreira do Amaral

**Keywords:** Endometriosis, Pelvic pain, Laparoscopy, Infertility, Histology, Endometriose, Dor pélvica, Laparoscopia, Infertilidade, Histologia

## Abstract

**CONTEXT AND OBJECTIVE:**

Diagnoses of endometriosis are based on observation of endometriotic lesions by means of laparoscopy, along with the pathological findings. The aim of this study was to evaluate the sensitivity and specificity of the macroscopic findings in relation to the histopathological findings. More specifically, we aimed to test the efficacy of laparoscopy alone for diagnosing endometriosis and to evaluate the laterality of endometriosis among the study population.

**DESIGN AND SETTING:**

Cross-sectional study on women undergoing laparoscopy due to pelvic pain or infertility, in the Gynecology Department of Hospital Santa Cruz in Curitiba, Paraná, Brazil, and Pontifícia Universidade Católica do Paraná.

**METHODS:**

A total of 976 patients underwent laparoscopy and biopsy due to pelvic pain and/or infertility. We analyzed the laparoscopic and histopathological findings from patients with pelvic endometriosis (n = 468) and patients without endometriosis (n = 508).

**RESULTS:**

In 468 (47.95%) of the cases, the clinical and laparoscopic findings were consistent with endometriosis, and this was confirmed histopathologically in 337 (34.5%). Among the remaining 508 patients, although the laparoscopy was performed for other reasons relating to acute pelvic pain, eight were diagnosed with endometriosis from histopathological examination of the pelvic specimens obtained. Therefore, endometriosis was confirmed in 345 patients (35.3%). In comparison with the histopathology, laparoscopy alone presented 97.68% sensitivity, 79.23% specificity, 72% positive predictive value and 98.42% negative predictive value.

**CONCLUSION:**

Laparoscopy should be used in conjunction with histopathology for diagnosing endometriosis.

## RESUMO

**CONTEXTO E OBJETIVO:**

O diagnóstico da endometriose é determinado pela visualização dos implantes à laparoscopia e pela comprovação histológica. O objetivo deste trabalho foi avaliar a sensibilidade e a especificidade dos achados macroscópicos cirúrgicos e histopatológicos. Avaliou-se a eficácia da laparoscopia isoladamente no diagnóstico da endometriose e a lateralidade da doença.

**TIPO DE ESTUDO E LOCAL:**

Estudo transversal realizado no Serviço de Ginecologia do Hospital Santa Cruz em Curitiba, Paraná e na Pontifícia Universidade Católica do Paraná.

**MÉTODOS:**

Foram avaliadas 976 pacientes submetidas à videolaparoscopia por dor pélvica ou infertilidade e a biópsia. Foram analisados os achados laparoscópicos e histológicos de 468 pacientes com endometriose pélvica e de 508 pacientes sem endometriose.

**RESULTADOS:**

Foram selecionadas 468 (47,95%) pacientes para inclusão no presente estudo por apresentarem quadro clínico e videolaparoscópico de suspeita de endometriose. As 508 (52,04%) pacientes restantes tiveram indicação da cirurgia por outras causas relacionadas à dor pélvica e oito tiveram o diagnóstico de endometriose pelo anatomopatológico. A endometriose foi confirmada em 345 pacientes (35,3%). Ao compararmos a análise histológica com os achados a videolaparoscopia, observou-se sensibilidade de 97,68%, especificidade de 79,23%, valor preditivo positivo de 72%, valor preditivo negativo de 98,42%.

**CONCLUSÃO:**

Laparoscopia deve ser usada em conjunto com histopatologia para o diagnóstico de endometriose.

## INTRODUCTION

Endometriosis is described as a benign disease of the female genital system. It is principally characterized by endometrium-like tissue, consisting of glands and/or stroma, found outside the uterine cavity. Although implanted ectopically, this tissue presents histopathological and physiological responses that are similar to the responses of the endometrium.^1^

The main symptom of endometriosis is pelvic pain, which is often very intense. Dysmenorrhea and other complaints like dyspareunia and infertility are also seen.^2,3^

The diagnostic hypothesis of endometriosis is based on the clinical history, along with the results from gynecological examinations, laboratory tests and transvaginal ultrasound.^4,5^ Some clinical characteristics, the physical examination itself, laboratory test results and evidence from imaging examinations may suggest the diagnosis.^6^ The greatest difficulty lies in diagnosing minimal and mild lesions. In these cases, the ideal access for confirmation is always laparoscopic, since the complementary examinations available do not offer the necessary specificity.^7^

Diagnosis by means of laparoscopy, which is considered the gold standard, may depend to an as yet unknown degree on confirmation by means of histopathological assessment. However, the definitive diagnosis of the disease can only be obtained through histopathological examination of the biopsy sample.^8^

Assessment of the accuracy of laparoscopy for diagnosing endometriosis has demonstrated that it is highly precise in ruling out the disease, thereby informing therapeutic decisions.^9^ Recent studies have shown that endometriosis is principally diagnosed by laparoscopy combined with histopathological examination, although a negative result does not rule out the possibility of the disease.^10^

## OBJECTIVE

The objective of this study was to assess the sensitivity and specificity of the macroscopic findings from laparoscopy, in relation to diagnoses of endometriosis based on the results from histopathological examinations. More specifically, we aimed to test the efficacy of laparoscopy alone for diagnosing endometriosis and to evaluate the laterality of endometriosis among the study population.

## MATERIALS AND METHODS

This was a cross-sectional study. We analyzed 976 women who underwent laparoscopy due to pelvic pain and/or infertility at the Obstetrics and Gynecology Department of Hospital Santa Cruz between 1994 and 2004. We analyzed the laparoscopic and histopathological findings from all the patients. Of these 976 patients, 468 presented pelvic endometriosis and 508 patients did not present endometriosis (but had other gynecological conditions).

This study was analyzed and approved by the Ethics Committee of Pontifícia Universidade Católica do Paraná (PUCPR), under Ethics Committee Registration No. 530 and protocol No. 056.476.

The criteria for performing laparoscopy were as follows: the subject needed to be in the menacme and presenting pelvic pain, dyspareunia, dysmenorrhea or infertility; and the results from complementary tests such as CA125 determination and ultrasound needed to reveal pelvic masses or blood in the pelvic cavity. Patients who had not yet reached the menarche or had reached the menopause and cases of laparoscopic reintervention were excluded from the laparoscopy performed due to pelvic pain.

During the laparoscopy, we performed biopsies on anatomical abnormalities that presented the macroscopic appearance of endometriosis, i.e. typical lesions such as "powder burn", of reddish color (light or dark), light color (yellow or brown) or dark color (black or blue), or even on fibrotic lesions. The lesions suggestive of endometriosis were biopsied and histopathologically examined in the Pathological Anatomy Department of Hospital Santa Cruz. The endometriosis was staged in accordance with the 1985 American Fertility Society (AFS) classification system, and the staging was compared with the result from the histopathological analysis on the biopsies.^11^

Pearson's chi-squared test and Fisher's exact test were used to assess any proportional differences between the groups with and without endometriosis. Differences between the continuous variables were studied using analysis of variance (ANOVA). The significance level was set at P ≤ 5% for all tests and the power test was 90%.

## RESULTS

**Figure 1:**
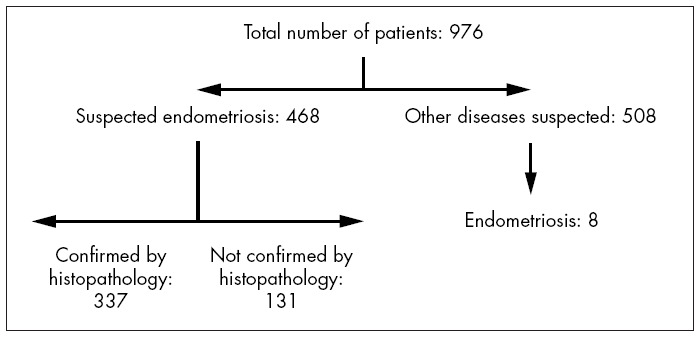
Flowchart of the distribution of the patients selected for this study.

Out of the 976 patients who underwent laparoscopy, 468 (47.95%) were selected for inclusion in the present study, since they presented clinical and laparoscopic profiles suggestive of endometriosis. Among these patients, the clinical and laparoscopic suspicion of endometriosis was confirmed from histopathological analysis in 337 cases (72.0%) (Figure 1).

In the cases of a further eight patients, a positive histopathological diagnosis was made during surgical procedures that were performed due to other causes. Therefore, a total of 345 patients were diagnosed through histopathology.

The mean age of these patients was 30.85 ± 5.54 years. The frequency of endometriosis of any stage was found to be highest among patients between the ages of 20 and 40 (P = 0.001).

The endometriosis was classified as follows: minimal (15 cases, 4.34%); mild (176 cases, 51.01%); moderate (112 cases, 32.46%); or severe (42 cases, 12.17%).

Out of the 345 patients evaluated, 341 (98.84%) presented acute or chronic pelvic pain. Acute pain was more common among the patients presenting the milder stages of endometriosis, whereas chronic pelvic pain was more common in the more severe stages (P = 0.03).

There were 129 patients (37.39%) who complained of dysmenorrhea, 69 (20%) who reported primary infertility and 23 (6.66%) who reported secondary infertility. A tendency towards higher frequency of dysmenorrhea was found among patients with the more severe forms of endometriosis, whereas the frequency of primary or secondary infertility was comparable at all stages of the disease (Table 1).

**Table 1:** Laparoscopic endometriosis staging, by patient age and clinical manifestation

	Minimal (stage I) (n = 15)	Mild (stage II) (n = 176)	Moderate (stage III) (n = 112)	Severe (stage IV) (n = 42)	P
Age (years)*	31.4 ± 6.74	30.78 ± 5.8	30.84 ± 5.15	30.97 ± 5.14	0.97
Chronic pelvic pain	3 (20%)	21 (11.93%)	26 (23.21%)	12 (28.57%)	0.03
Acute pelvic pain	12 (80%)	155 (88.06%)	86 (76.78%)	32 (76.19%)	0.07
Dysmenorrhea	5 (33.33%)	59 (33.52%)	44 (39.28%)	21 (50%)	0.23
Primary infertility	3 (20%)	34 (19.31%)	27 (24.1%)	05 (11.9%)	0.39
Secondary infertility	1 (6.66%)	15 (8.52%)	7 (6.25%)	0 (0%)	0.26

* *Age expressed as mean ± standard deviation; all other values expressed as number and percentage.*

The histopathological examination confirmed the presence of endometriosis in the right ovary in 77 cases (22.31%) and in the left ovary in 89 cases (25.79%). No statistically significant difference in frequency was observed between the right and left ovaries (22.31% versus 25.79%, P > 0.05). In 29 patients (8.4%), both ovaries were involved.

Endometriosis was identified in the peritoneum in 260 patients (75.36%) and in the rectovaginal septum in 41 (11.88%).

Endometriosis was confirmed in only one of the biopsied sites in 233 patients (67.53%), in two sites in 102 (29.56%), in three sites in eight (2.31%) and in four sites in two (0.57%). The laparoscopic analysis suggested a diagnosis of minimal endometriosis in 17 patients (12.97%), mild endometriosis in 63 (48.09%), moderate endometriosis in 35 (26.71%) and severe endometriosis in 16 (12.21%).

Taking the histopathological findings to be definitive for the diagnosis of endometriosis, the clinical suspicion and laparoscopic findings presented 97.68% sensitivity, 79.23% specificity, 72% positive predictive value, 98.42% negative predictive value, and 85.75% accuracy (Table 2). False positive results were obtained in 27.99% of the tests, compared with false negative results in 1.57% of the tests.

**Table 2:** Laparoscopic and histopathological findings (n = 976)

Surgical diagnosis (laparoscopy)	Histopathological confirmation		Total
	Positive	Negative	
Positive	337 (34.52%)	131 (13.42%)	**468 (48.15%)**
Negative	8 (0.81%)	500 (51.22%)	**508 (52.04%)**
**Total**	**345 (35.34%)**	**631 (64.65%)**	**976 (100%)**

## DISCUSSION

Despite the efforts of the scientific community to increase the efficacy of the methods used to diagnose endometriosis, various limitations remain, thus making it difficult to reach a definitive diagnosis.

Clinical parameters such as pelvic pain, dysmenorrhea, dyspareunia and infertility are insufficient to confirm the diagnosis. Likewise, combining laboratory tests such as CA125 level determinations with imaging methods such as ultrasonography, tomography and magnetic resonance provides relative value for reaching a conclusive diagnosis in the initial stages of endometriosis.^12-14^ Combining laparoscopy with histopathological examination yields greater sensitivity for the definitive diagnosis of the disease and also decreases the diagnostic errors.^15^

Among the 976 laparoscopies performed in this study, the frequency of endometriosis was 35.3%. This result is in accordance with findings from previous studies carried out among smaller population samples.^16,17^ Furthermore, our findings corroborate data in the literature regarding the mean age of the patients studied and are in keeping with the results from other studies showing that the onset of symptoms usually occurs within seven to twelve years after the menarche.^18^

In the present study, and in accordance with the 1985 AFS system for staging endometriosis,^12^ 4.36% of our patients were classified as stage I (minimal), 51.01% as stage II (mild), 32.46% as stage III (moderate) and 12.17% as stage IV (severe). In a study involving 44 patients who underwent laparoscopy due to pelvic pain, Petta reported that 50% presented stage I endometriosis, 12.5% presented stage II, 25% presented stage III and 12.5% presented stage IV.^19^ These results, together with others found in the literature, are listed in Table 3.^19,20-22^

**Table 3:** Staging of endometriosis in the literature

Authors	Stage I (minimal)	Stage II (mild)	Stage III (moderate)	Stage IV (severe)
Petta et al.^19^ (n = 44)	50%	12.5%	25%	12.5%
Gruppo Italiano per lo Studio dell´ Endometriosi.^20^ (n = 469)	11.3%	12.2%	51%	21.7%
Bai et al.^21^ (n = 39)	10%	44%	28%	18%
Chapron et al.^22^ (n = 209)	13.5%	38.1%	24.2%	24.2%
Almeida Filho, Oliveira & Amaral (current study) (n = 345)	4.3%	51%	32.4%	12.1%

Between these different studies, discrepancies can be observed among the stages found. Although one particular macroscopic mapping method for endometriosis was recommended by the American Society for Reproductive Medicine in 1997, the results from many studies differ according to the background and experience of the professional who performed the laparoscopy. Therefore, comparative assessments are affected.^20^ The results from the present study demonstrate the difficulties ensuing from macroscopic assessments made by various observers. The diversity of the results justifies the use of histopathological analysis for diagnostic confirmation of endometriosis.^23^

The diagnosis of histopathology-confirmed endometriosis presented a statistically significant association with chronic pelvic pain. However, according to the findings of Wardle and Hull,^24^ acute pelvic pain, dysmenorrhea, primary infertility and secondary infertility had no statistically significant influence on the diagnosis of endometriosis.

To date, there is no consensus on the relationship between the extent of endometriosis and the intensity of pelvic pain.^25^ It has been shown that there is a correlation between certain histopathological findings (a well-differentiated pattern or a diagnosis of stromal disease) and the intensity of pelvic pain.^26^ In the present study, 98.84% of all patients (regardless of endometriosis stage) reported pelvic pain. Pelvic pain was found to correlate significantly with endometriosis stage (P = 0.03) (Table 3).

In other studies, it was reported that the severity of dysmenorrhea presented no significant association with the stage or location of endometriosis.^20,27^ Our results are in accordance with those of such studies, in that no positive correlation was found between the degree of endometriosis and the intensity of dysmenorrhea.^27^ We observed dysmenorrhea in 37.39% of our patients with confirmed endometriosis and in 26.71% of our patients without endometriosis, although the difference was not statistically significant.

Topalski Fistes et al.^27^ carried out a comparative study with a control group of 200 fertile women. They found that the frequency of endometriosis was 32% among infertile women and 5% among fertile women, which was a statistically significant difference (P = 0.001). In the present study, the frequencies of primary or secondary infertility were comparable, regardless of the severity of the disease.

When we compared the laterality of ovarian involvement in the 345 women evaluated, we found similar frequencies (left ovary versus right ovary: 25.79% versus 22.31%; P > 0.05). Several studies evaluating endometriotic ovarian cysts have shown a predisposition towards left-sided lesions.^8,28,29^ However, this was not confirmed in our study.

In addition, we observed a greater incidence of the disease in the peritoneum (79.3%), regardless of the stage of endometriosis, whereas the incidence of peritoneal lesions described in the literature ranges from 17.5% to 31%.^19,20,22^

In the present study, the number of biopsies testing positive for endometriosis was directly proportional to the severity of the endometriosis. This shows that, whether laparoscopy or histopathology is used, it is more difficult to make a definitive diagnosis when the lesions are minimal or mild.

In a study assessing macroscopic findings of anatomical abnormalities and confirmation of endometriosis, it was found that 85.7% of the patients presented pelvic anatomical abnormalities consistent with endometriotic lesions and that 31.1% of them were identified through histopathology as endometriosis.^20^ In our study, 468 patients presenting pelvic pain and anatomical abnormalities typical of endometriosis were evaluated, and the diagnosis of endometriosis was confirmed in 337 (72%).

Comparison between these studies reveals that, despite the validity of laparoscopy for diagnosing endometriosis, its use without histopathological confirmation gives rise to discrepancies in relation to the macroscopic findings.^10^ There is a need for an informal consensus regarding study design, and good surgical practice should be supported by detailed documentation in order to systematize the diagnosis.^10^

The findings from the present study allow us to conclude that endometriosis demonstrated a significant positive correlation with chronic pelvic pain, although not with dysmenorrhea or infertility. A greater frequency of peritoneal endometriosis was observed, in comparison with the involvement of other sites, such as the rectovaginal septum or ovaries.

## CONCLUSIONS

The results obtained suggest that laparoscopy alone is of limited efficacy. Therefore, it needs to be combined with histopathological examination in order to achieve diagnostic confirmation of endometriosis. Among the cases of ovarian endometriosis, there was no difference in laterality.
